# Doped Graphene for DNA Analysis: the Electrochemical Signal is Strongly Influenced by the Kind of Dopant and the Nucleobase Structure

**DOI:** 10.1038/srep33046

**Published:** 2016-09-14

**Authors:** Huidi Tian, Lu Wang, Zdenek Sofer, Martin Pumera, Alessandra Bonanni

**Affiliations:** 1 Division of Chemistry & Biological Chemistry, School of Physical and Mathematical Sciences, Nanyang Technological University, 637371, Singapore; 2 Department of Inorganic Chemistry, Institute of Chemical Technology, 166 28 Prague 6, Czech Republic

## Abstract

Doping graphene with heteroatoms can alter the electronic and electrochemical properties of the starting material. Contrasting properties should be expected when the doping is carried out with electron donating species (n-type dopants) or with electron withdrawing species (p-type dopants). This in turn can have a profound influence on the electroanalytical performance of the doped material being used for the detection of specific probes. Here we investigate the electrochemical oxidation of DNA bases adenine, guanine, thymine and cytosine on two heteroatom-doped graphene platforms namely boron-doped graphene (p-type dopant) and nitrogen-doped graphene (n-type dopant). We found that overall, boron–doped graphene provided the best response in terms of electrochemical signal sensitivity for all bases. This is due to the electron deficiency of boron-doped graphene, which can promote the oxidation of DNA bases, as opposed to nitrogen-doped graphene which possesses an excess of electrons. Moreover, also the structure of the nucleobase was found to have significant influence on the obtained signal. Our study may open new frontiers in the electrochemical detection of DNA bases which is the first step for label-free DNA analysis.

Heteroatom-doped graphene has recently been the focus of several studies due to its exceptional structural, physicochemical, electrical and electrochemical properties^1^,^2^,^3^,^4^,^5^,^6^,^7^. The doping of graphene materials either with electron donating or electron withdrawing species results in a significant change in the electron density of graphene sheets^8^. This in turn may have a strong influence on the electrochemical properties of the obtained material^9^,^10^. It should be expected that contrasting electronic and electrochemical properties were shown when doping the graphene with n-type dopants (electron donors) such as nitrogen, as compared to p-type dopants (electron acceptors) such as boron^11^,^12^. However, recent studies have shown that both nitrogen-doped graphene and boron-doped graphene are able to provide an improved electrochemical performance for the detection of common biomarkers. For instance, an enhanced sensitivity in the electrochemical detection of ascorbic acid, dopamine and uric acid was shown when using nitrogen-doped graphene platforms^9^,^13^; likewise, a boron and nitrogen co-doped graphene platform showed increased sensitivity in the detection of H_2_O_2_ released from leukemia cells^14^; at the same time, an increased sensitivity, selectivity and linearity of response was shown by boron-doped graphene for the electrochemical detection of gallic acid^15^. From the literature, it seems clear that depending on the structure of the analyte, different kinds of dopant may promote the interactions between the former and the heteroatom doped graphene, thus improving the electrochemical performance^16^. Thus far, only few studies focused on specific probes and compare the analytical performance of n-type and p-type doped graphenes for their detection. Hence, there is an urgent need to extend the investigation field in order to compare and select the heteroatom-doped graphene material which is better suited to the specific aim.

Here we investigate the electrochemical oxidation of nucleobases namely adenine, guanine, thymine and cytosine^17^,^18^,^19^,^20^,^21^,^22^,^23^ using one p-type and one n-type heteroatom-doped graphene. The n-type material was obtained by thermally exfoliating graphite oxide in the presence of a nitrogen precursor, whilst a boron precursor was used in the synthesis of p-type doped graphene.

We explore here how the oxidation of DNA bases is influenced by the presence of the heteroatom, and whether the material properties and the specific base structure play a role in the electrochemical detection. To the best of our knowledge, this is the first work in which the electrochemical behaviour of DNA bases is compared on different heteroatom-doped graphene platforms. We found that the kind of heteroatom and the structure of DNA bases are the parameters that mostly influence the electrochemical oxidation of the latter, rather than the structural properties of the materials.

## Experimental

### Materials

Graphite was purchased from Asbury Carbons. Fuming nitric acid (>90%) was provided by J.T. Baker. Hydrogen peroxide (3%), sulfuric acid (95–98%), potassium chlorate (98%), *N,N-*dimethylformamide (DMF), hydrochloric acid (37%), ethanol, sodium chloride, sodium hydroxide, potassium phosphate dibasic, sodium phosphate monobasic, sodium nitrate, thiourea dioxide, potassium permanganate, potassium chloride, adenine hydrochloride (A), guanine hydrochloride (G), cytosine (C) thymine (T) and were obtained from Sigma-Aldrich (Singapore). The glassy carbon (GC) electrodes with 3 mm diameter, Ag/AgCl electrode and platinum (Pt) electrode were obtained from CH Instruments, TX (US).

### Apparatus

Electrochemical measurements were carried out by using a μAutolab type III electrochemical analyzer (Eco Chemie, The Netherlands) controlled by General Purpose Electrochemical Systems Version 4.9 software (Eco Chemie). The applied parameters were as follows: accumulation time at 0.2 V: 60 s; modulation time: 50 ms; interval time: 0.5 s; modulation amplitude: 25 mV; step: 5 mV. Baseline correction with a peak width of 0.01 was applied to raw data. Electrochemical experiments were performed at room temperature (25 °C) in an 8 mL voltammetric cell using a three-electrode system. A platinum electrode served as a counter electrode; an Ag/AgCl electrode served as a reference electrode.

X-ray photoelectron spectroscopy (XPS) was performed by using a monochromatic Mg X-ray radiation source (SPECS, Germany) and a Phoibos 100 spectrometer. Raman spectra were measured by using a confocal micro-Raman LabRam HR instrument (Horiba Scientific) in backscattering geometry with a CCD detector with an Ar laser at 514.5 nm. A 100 × objective lens, mounted on an Olympus optical microscope, was used for the measurement. The calibration was performed with a silicon reference with a peak position at 520 cm^−1^ and the resolution was less than 1 cm^−1^.

A JEOL 7600 field emission scanning electron microscope (SEM) was employed to obtain scanning electron micrographs of the materials.

### Protocols

#### Synthesis of nitrogen-doped graphene (N-G)

N-G was prepared by thermal exfoliation of graphite oxide prepared by Hummers method, by following a previously optimized protocol^24^,^25^. The obtained graphite oxide (100 mg) was placed into a quartz glass reactor. Before inserting the sample into the hot zone, the reactor was repeatedly evacuated and flushed with N_2_. After that the N_2_ flow was switched to NH_3_ with a flow rate of 300 mL/min and constant T of 600 °C for 12 min.

#### Synthesis of boron-doped graphene (B-G)

B-G was prepared by thermal exfoliation of graphite oxide prepared by Staudenmaier method, by following a previously optimized protocol^26^,^27^. The prepared graphite oxide was exfoliated in the presence of boron trifluoride diethyl etherate (BF_3_,Et_2_O) used as boron precursor. The carrier gas was N_2_ diluted with 1 L/min of N_2_ and a H_2_/N_2_ mixture (0.5 L/min N_2_ and 0.5 L/min H_2_). The reactor was kept flushed with nitrogen with flow rate of 100 mL/min. Before moving the sample to the hot zone of the reactor, the flow of boron precursor was stabilized for 5 minutes. After that, the exfoliation was performed at 1000 °C for 12 minutes.

Glassy carbon (GC) electrode was renewed by polishing with 0.05 μm alumina powder before using. Boron-doped graphene (B-G) powder (5 mg ml^−1^) and nitrogen-doped graphene (N-G) powder (5 mg ml^−1^) were dispersed in DMF. Before use, the dispersions were ultrasonicated for few minutes. 1 μL of the dispersion was dropcasted onto a GC electrode. The DMF solvent was then allowed to evaporate under a lamp in order to leave a randomly distributed film on GC surface. Electrochemical experiments were performed in an 8 mL voltammetric cell at room temperature (25 °C) using a three-electrode system. When B-G was used to measure cytosine and thymine, a pre-treatment of 5 voltammetric scans from 0 V to 1.8 V was applied to the material. Same pre-treatment was applied to N-G before measurement of all bases. All measurements were performed in 50 mM phosphate buffer (pH 7.2).

#### Scan rate studies

B-G and N-G electroactive surface area was calculated by Randles–Sevcik equation by measuring the peak intensity of 1 mM K_3_[Fe(CN)_6_] in 0.1 M KCl at different scan rates. The diffusion constant value was obtained from the literature (D = 7.2 × 10^−6^ cm^2^s^−1^)^28^.

In addition, a further scan rate study was performed to measure the oxidation peak of all DNA bases on B-G and N-G by using the following scan rates: 25, 50, 75, 100, 125, 150, 200 mV.

## Results and Discussion

**Figure 1:**
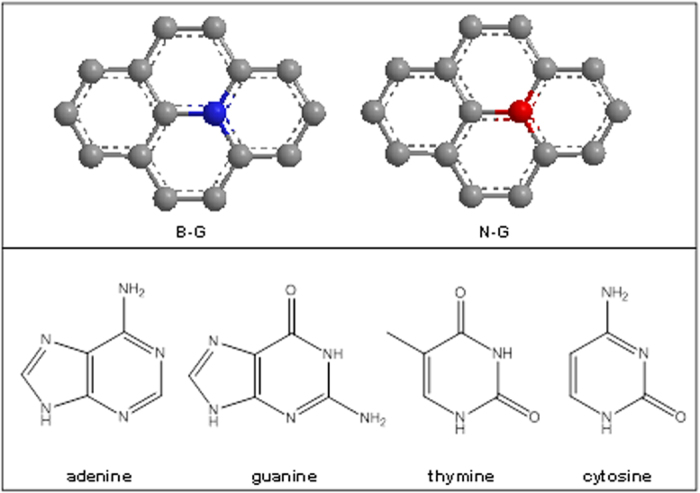
Models for boron-doped graphene (B-G), nitrogen-doped graphene (N-G), and chemical structure of DNA nucleobases.

The heteroatom doped graphenes used in this study were obtained by thermal exfoliation of graphite oxide, either in the presence of the boron precursor (i.e. BF_3_) or in the presence of the nitrogen precursor (i.e. NH_3_). Given the experimental conditions, substitutional doping occurs in the graphene lattice, as shown in Fig. 1. This lead to the formation of p-type material due to the presence of electron deficient boron (B-G), and n-type material due to the presence of electron rich nitrogen (N-G).

**Figure 2:**
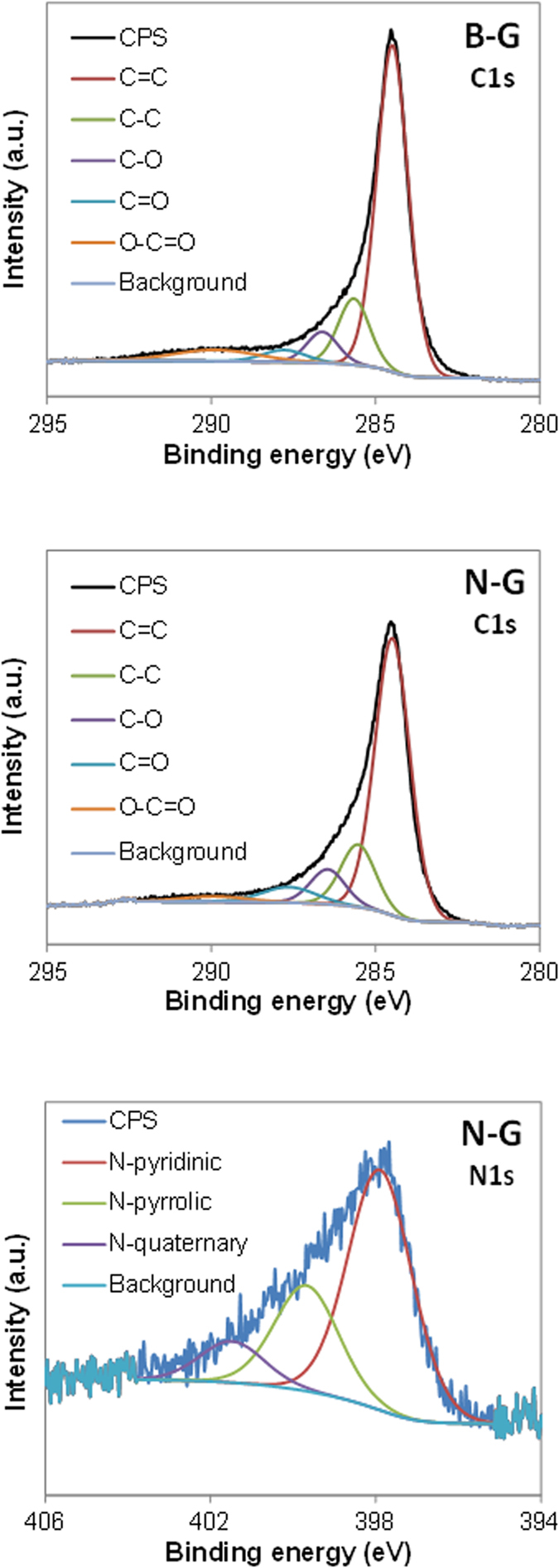
C1s high resolution X-ray photoelectron spectra for boron-doped graphene (B-G) and nitrogen-doped graphene (N-G) and N1s high resolution X-ray photoelectron spectra for nitrogen-doped graphene (N-G).

The materials were characterized by prompt gamma-activation analysis^27^, XPS, SEM and Raman spectroscopy. The characterization performed on boron-doped graphene (B-G) revealed that the amount of boron was 23 ppm whilst the level of doping in the nitrogen-doped graphene (N-G) was 6.4 at.% N. From XPS characterization B-G showed a higher C/O ratio of 13.83 ± 0.52 as compared to C/O ratio of 12.34 ± 0.76 shown by N-G, indicating that a lower amount of oxygen functionalities are present on B-G surface (see Fig. 2, part A and B for C1s high resolution spectra of B-G and N-G respectively). The boron in B-G could not be detected by XPS at 190 eV due to low doping level (note that the detection limit of XPS is ~0.1 atom % and the amount of B is far below this value)^29^, for this reason a more sensitive technique namely prompt gamma-activation analysis was employed for the determination of the amount of boron in B-G material^27^. On the other end, a clear signal for nitrogen was recorded around 400 eV (see Fig. 2 part C for N1s high resolution spectra of N-G) which confirmed the presence of pyrrolic, pyridinic and quaternary nitrogen in the graphene network.

**Figure 3:**
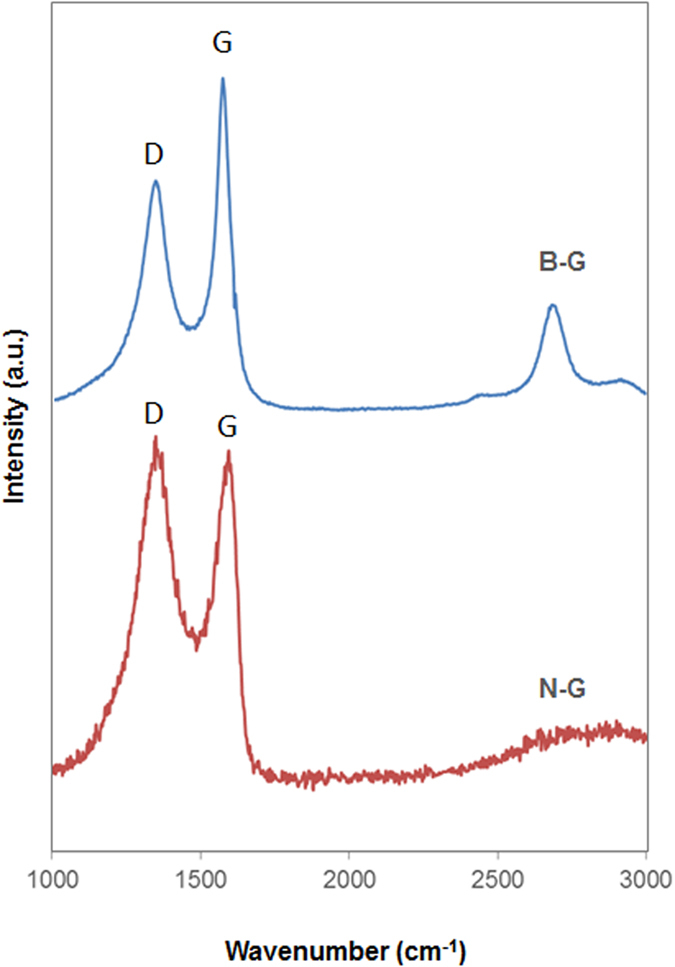
Raman spectra of boron-doped graphene (**B-G**), and nitrogen-doped graphene (N-G). Spectra were normalized with respect to G band for a clearer comparison.

The D/G ratio measured by Raman spectroscopy is correlated to the density of defects that can be found in the sp^2^ network. From Raman characterization B-G displayed a D/G ratio of 0.79 ± 0.16 which is lower than that of N-G at 1.03 ± 0.01, revealing that a lower degree of disorder is present on B-G surface as compared to N-G (see Fig. 3). As it can also be observed in the figure, the 2D-signal around 2720 cm^−1^ is lacking in the spectrum of N-G, indicating the presence of significant structural defects^30^.

The morphology of both B-G and N-G was studied by SEM, as shown in Figure S1 of Supporting Information. A characteristic exfoliated structure was presented by both materials, thus confirming the successful thermal exfoliation of graphite oxide in the presence of BF_3_ and NH_3_ atmosphere respectively^25^,^27^. The electroactive surface area was estimated by performing a scan rate study of the materials in the presence of K_3_[Fe(CN)_6_]. The results showed that the electroactive surface area of N-G was (8.62 ± 0.0814) ×10^−2 ^cm^2^ which is almost double as compared to that of B-G at (4.72 ± 0.0580) ×10^−2 ^cm^2^. For the complete data from the study please refer to Figure S2 of Supporting Information.

The materials were then employed as electrochemical platform for the oxidation of DNA nucleobases.

**Figure 4:**
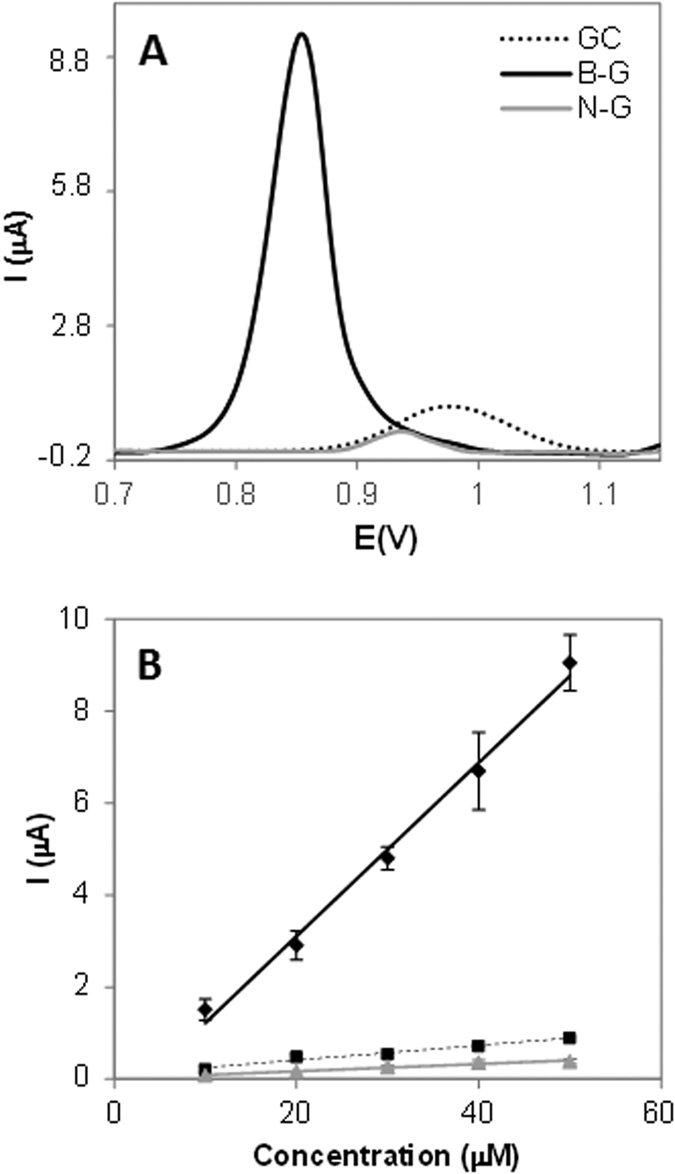
DPV study of adenine on GC, B-G, and N-G modified electrodes in 50 mM phosphate buffer solution (pH 7.2). (**A**) Peak current of adenine at 50 μM concentration; (**B**) Peak current vs concentration of adenine. Experimental conditions for DPV: accumulation time 0.2 V: 60 s; modulation time: 50 ms; interval time: 0.5 s; modulation amplitude: 25 mV; step: 5 mV; potential range: 1–1.2 V; scan rate: 20 mV. Peak current intensities for calibration curves are obtained from triplicate experiments.

**Figure 5:**
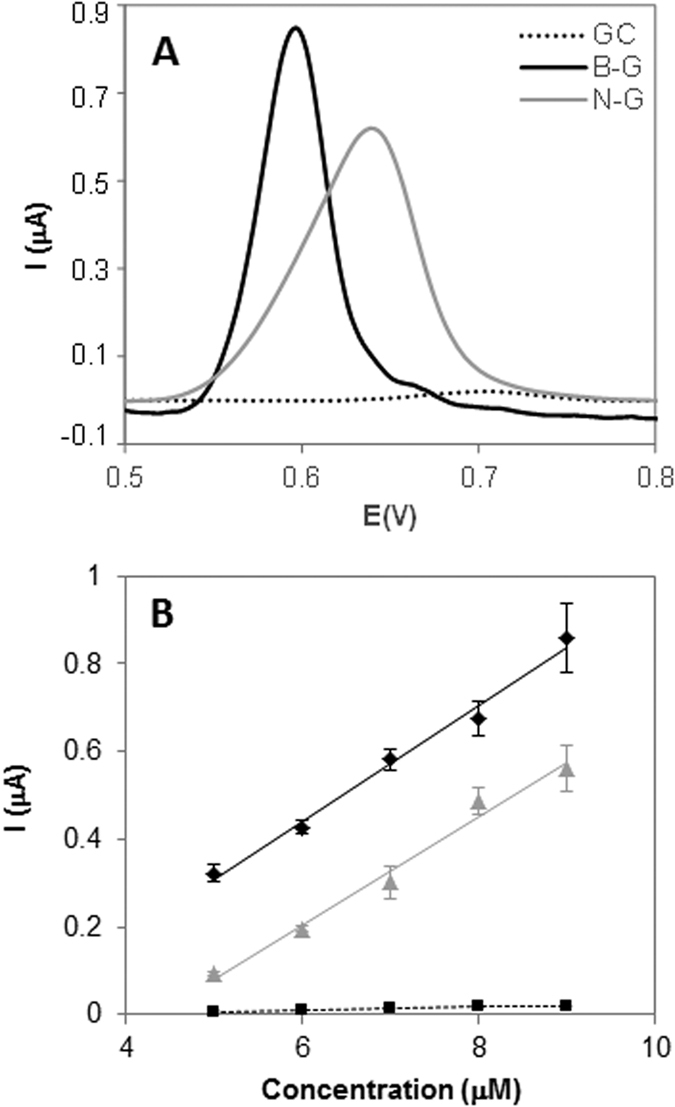
DPV study of guanine on GC, B-G, and N-G modified electrodes in 50 mM phosphate buffer solution (pH 7.2). (**A**) Peak current of guanine at 9 μM concentration; (**B**) Peak current vs concentration of guanine. Experimental conditions for DPV: accumulation time 0.2 V: 60 s; modulation time: 50 ms; interval time: 0.5 s; modulation amplitude: 25 mV; step: 5 mV; potential range: 1–1.2 V; scan rate: 20 mV. Peak current intensities for calibration curves are obtained from triplicate experiments.

**Figure 6:**
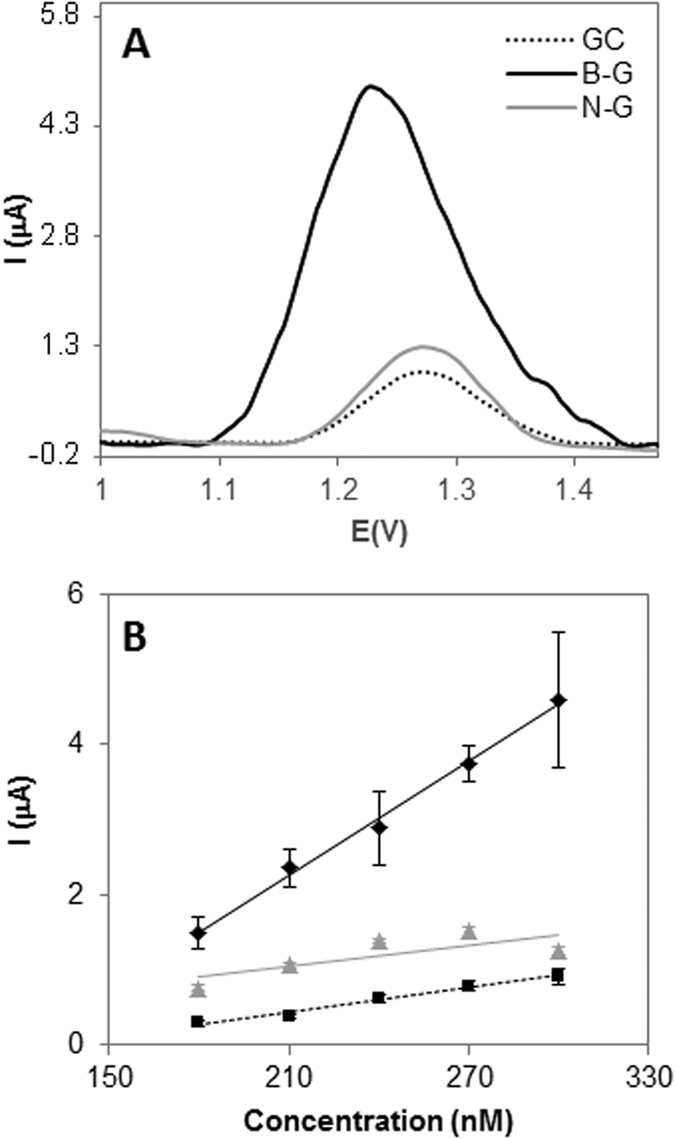
DPV study of thymine on GC, B-G, and N-G modified electrodes in 50 mM phosphate buffer solution (pH 7.2). (**A**) Peak current of thymine at 100 nM concentration; (**B**) Peak current vs concentration of thymine. Experimental conditions for DPV: accumulation time 0.2 V: 60 s; modulation time: 50 ms; interval time: 0.5 s; modulation amplitude: 25 mV; step: 5 mV; potential range: 1–1.2 V; scan rate: 20 mV. Peak current intensities for calibration curves are obtained from triplicate experiments.

**Figure 7:**
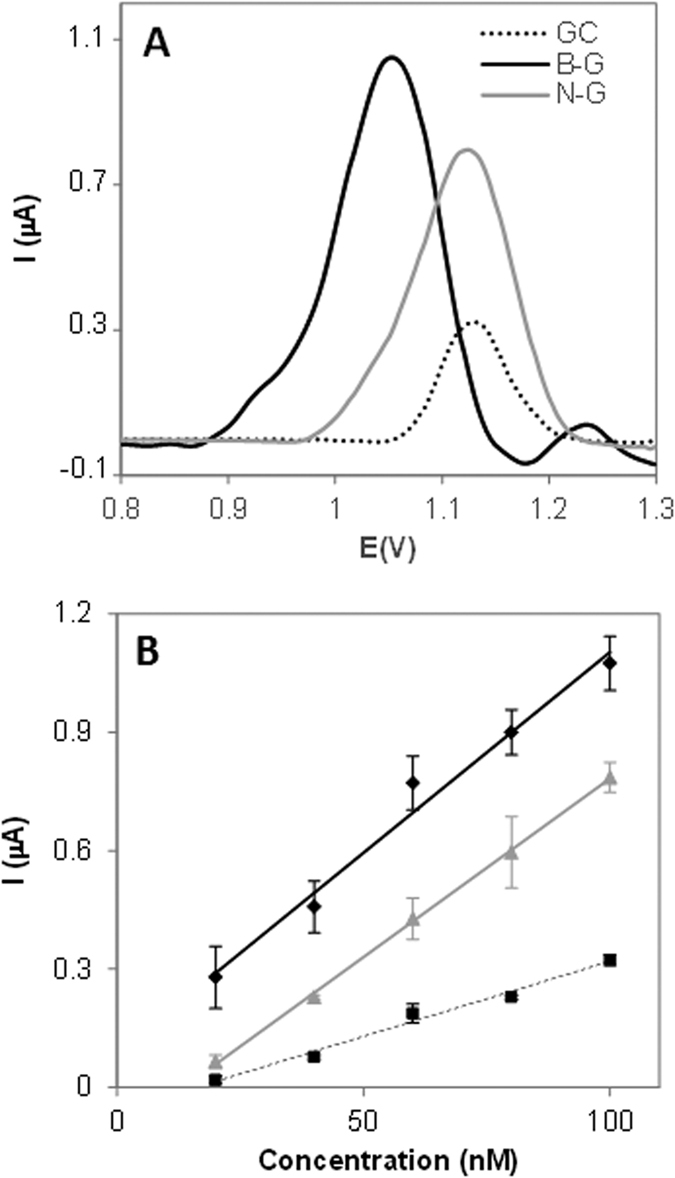
DPV study of cytosine on GC, B-G, and N-G modified electrodes in 50 mM phosphate buffer solution (pH 7.2). (**A**) Peak current of cytosine at 270 nM concentration; (**B**) Peak current vs concentration of cytosine. Experimental conditions for DPV: accumulation time 0.2 V: 60 s; modulation time: 50 ms; interval time: 0.5 s; modulation amplitude: 25 mV; step: 5 mV; potential range: 1–1.2 V; scan rate: 20 mV. Peak current intensities for calibration curves are obtained from triplicate experiments.

Figure 4 shows the DPV peaks and calibration curves obtained by representing the oxidation peak height versus the concentration of adenine on three different platforms namely GC (black dotted line), B-G (black continuous line), and N-G (grey continuous line) modified electrodes. In the same way the results obtained for guanine, thymine and cytosine oxidation are displayed in Figs 5, 6 and 7. Peak current intensities for calibration curves were obtained from triplicate experiments.

Table 1 displays the analytical parameters such as calibration sensitivity, repeatability, linearity and selectivity of response for DNA nucleobase oxidation on the three platforms. Concentration ranges chosen for the study were optimized in a previous study in which the linearity of response for each nucleobase on graphene materials was carefully assessed^18^.

Overall, it can be observed that B-G showed better sensitivity in the electrochemical signal (indicated by a larger slope in the calibration curve which corresponds to the calibration sensitivity) than N-G for all DNA bases. This can be explained by considering that during the oxidation of DNA bases electrons are withdrawn by the electrochemical platform; it is then expected that a platform containing electron deficient heteroatom such as boron in B-G would have a favourable effect on the oxidation of DNA bases as compared to electron donating nitrogen in N-G. Moreover, the presence of boron also facilitated the electron transfer, making the oxidation occurring at lower potentials for all bases, as shown in part A of Figs 4, 5, 6 and 7.

These results would be unexpected if we were focusing only on the material properties such as structural disorders and electroactive surface area, which indicate N-G as the material that should provide the best analytical performance, being richer in structural defects^31^,^32^,^33^ and showing a larger electroactive surface area as compared to B-G. In fact, when oxidation signal of DNA bases is represented in terms of current density instead of current intensity (see Figure S3, Supporting Information) which takes into account the electroactive surface areas of the materials, an even more pronounced difference is observed between B-G and N-G. Hence, the predominant effect on the analytical performance of heteroatom doped graphene for the oxidation of DNA bases must be due to the nature of the heteroatom rather than the structural features of the material.

This is reflected also in the different behaviour of the four DNA bases on B-G and N-G. In fact, it was observed that among the four bases adenine was the one showing the most prominent difference for oxidation occurring on B-G and N-G modified electrodes, followed by cytosine in which there was still an improved signal for B-G. As for guanine and thymine even though a larger signal was recorded on B-G as compared to N-G when using the same base concentration, for the calibration sensitivity not much difference was observed on the two materials. In order to explain the very different behaviour of adenine as compared to the other three DNA bases, the structure of the nucleobases should be taken into account (see Fig. 1).

From Fig. 1 it can be noticed that all DNA bases with exception of adenine possess the electron withdrawing group C=O, which presence may explain the observed electrochemical behaviour. In fact, this group can be favouring the interaction of DNA bases with N-G containing the electron donating nitrogen. As such, the oxidation of guanine, thymine and cytosine on N-G may be favoured by the presence of C=O group and this can explain the more similar behaviour of N-G to B-G for these three bases. On the other end, the favourable interaction with N-G was not observed for adenine due to the absence of C=O group. For this reason a more pronounced difference in the sensitivity of the electrochemical signal between B-G and N-G was observed on adenine.

In order to investigate the mechanism for the electrochemical oxidation of DNA nucleobases at doped graphene surfaces, a scan rate study was performed by varying the scan rate while recording the oxidation peak of DNA nucleobases at a fixed concentration^34^. The peak current was then plotted both versus the scan rate and the square root of the scan rate in order to understand if the oxidation process was diffusion-controlled or adsorption-controlled^35^. Overall, the study revealed that the electrochemical oxidation of DNA nucleobases on both doped graphenes can be considered neither a fully diffusion-controlled nor a totally adsorption-controlled process, but it is indeed a combination of both phenomena. However, when comparing the linearity of the peak current with either the scan rate or the square root of the scan rate, it can be assumed that the adsorption-controlled process is predominant as compared to the diffusion-controlled process (see R^2^ for all nucleobases on B-G and N-G depicted in Table S2). This confirms our hypothesis on the influence of C=O groups in guanine, cytosine and thymine for the detection on N-G materials, which can explain the more similar behaviour of N-G to B-G for these three bases. On the other end, the favourable interaction with N-G was not observed for adenine due to the absence of C=O group. For this reason a more pronounced difference in the sensitivity of the electrochemical signal between B-G and N-G was observed on adenine.

Being adenine the base showing the most pronounced difference for oxidation occurring on B-G and N-G modified electrodes, a further study was performed by comparing adenine oxidation signal on B-G, N-G and undoped graphene platform. In this study shown in Figure S4 (Supporting Information), the undoped graphene showed a lower electrochemical signal for adenine oxidation as compared to the electron withdrawing B-G platform, and a higher electrochemical signal when compared to the electron donating N-G platform.

In terms of repeatability of results, all materials showed good repeatability, being the RSD% values between 3.4% and 15.7%. The R^2^ can be correlated to the linearity of the analytical response. Overall, B-G was the material showing the best linearity of response with R^2^ included between 0.9815 and 0.9944, whilst a lower linearity in the analytical response was shown by N-G, especially in the case of cytosine in which a reduced linear dynamic range was observed. The peak width at half height can be associated to the selectivity of the response towards the single DNA base, being the smallest value indicative of the best selectivity. From the obtained results it can be noticed that B-G showed the best selectivity for the analysis of purines (i.e. guanine and adenine) whilst for pyrimidines (i.e. thymine and cytosine) the best selectivity was shown by GC. In addition, the limit of detection (LOD) for all bases on both B-G and N-G was evaluated by considering the standard error of the regression (residual standard deviation of the regression s_y/x_) and the calibration sensitivity (corresponding to the slope of the regression line)^36^. This method allows controlling both false positive error and false negative error at about 5% 37,38. The results showed an improved LOD on B-G for adenine, guanine and cytosine at 5.28 μM, 0.59 μM and 13.55 nM respectively, as compared to those obtained on N-G for the same bases at 10.30 μM, 0.73 μM and 147.35 nM respectively. Only for the detection of thymine an improved LOD was shown on N-G at 3.27 nM as compared to 16.51 nM which was obtained on B-G material. This last result could be due to the similar value of calibration sensitivity of thymine on both B-G and N-G and to the better reproducibility of results achieved with N-G material. In fact, the standard error of the regression for thymine was about 5 times higher on B-G as compared to that of N-G, and this can help explaining the different value obtained for the limit of detection on the two graphene materials.

In order to evaluate the applicability of B-G material to DNA analysis in real samples, an additional study was performed by measuring the oxidation signal of a mixture of all DNA bases on B-G. The results, shown in Figure S5 of Supporting Information, showed four distinct peaks belonging to guanine (at 0.62 V), adenine (at 0.91 V), thymine (at 1.10 V), and cytosine (at 1.27 V) respectively. The same experiment was performed as control on GC electrode. As clearly shown in Figure S5, a significant improvement both in the sensitivity of the signal and in the selectivity of the determination was achieved on B-G, while lower and lesser peaks are observable on GC platform. This demonstrated that our method allows the simultaneous detection of four DNA bases contained in a real DNA sample.

Finally, a stability study was performed by carrying out repeated measurements of adenine oxidation signal on the same B-G modified electrode. The results shown in Table S1 demonstrated a stable platform in which the signal decrease was still within the experimental error after 5 cycles (with 89% of initial signal recovered) and the 64% of initial signal could be still recovered after 20 DPV cycles.

## Conclusions

In this work one p-type and one n-type heteroatom doped graphene namely boron doped graphene (B-G) and nitrogen-doped graphene (N-G) were employed as platforms for the electrochemical detection of the nucleobases adenine, guanine, thymine, and cytosine.

From the material point of view, we can conclude that in general B-G provided the best calibration sensitivity for all bases due to the thermodynamically favourable electron transfer between the nucleobase and the electron withdrawing boron during the oxidation of the former. This favourable interaction was not observed on N-G due to the presence of electron donating nitrogen; for this reason a lower oxidation signal was recorded on this material, thus resulting in a lower value of the calibration sensitivity. Moreover, it is worthy of note that a lower oxidation potential is shown by DNA bases on B-G material, which in turn improves the selectivity when analysing DNA sequences.

From the nucleobase point of view, it was observed that the difference in the electrochemical signal obtained on B-G and N-G is strongly influenced by the nucleobase structure. The absence of the electron withdrawing carbonyl group on adenine, which could have favoured the interaction with the electron donating nitrogen during the oxidation on N-G, may account for the lower oxidation signal obtained on the latter. This in turn is reflected in the significant difference in the calibration sensitivities between B-G and N-G observed mainly for adenine oxidation.

Overall, it can be concluded that the oxidation of DNA bases on heteroatom doped graphene is strongly influenced by the kind of dopant and the nucleobase structure, rather than depending on the material structural features such as amount of defects and surface area. These findings may be important for the choice of the proper graphene platform for the electrochemical analysis of DNA by using compact devices based on the detection of DNA hybridization.

## Additional Information

**How to cite this article**: Tian, H. *et al*. Doped Graphene for DNA Analysis: the Electrochemical Signal is Strongly Influenced by the Kind of Dopant and the Nucleobase Structure. *Sci. Rep.* **6**, 33046; doi: 10.1038/srep33046 (2016).

## Supplementary Material

Supplementary Information

## Figures and Tables

**Table 1:** Calibration sensitivity (slope), relative standard deviation (RSD%), correlation coefficient (R^2^) and peak width at half height of DPV determinations of DNA nucleobases on GC, B-G, and N-G modified electrodes.

Nucleobase	Material	Slope/μA μM^−1^	RSD%	R^2^	*W*_1/2_/mV
adenine	GC	0.0159	9.9	0.9743	103
	B-G	0.1886	10.0	0.9923	55
	N-G	0.0079	15.2	0.9714	106
guanine	GC	0.0032	9.0	0.9934	71
	B-G	0.1321	5.8	0.9903	40
	N-G	0.1226	7.4	0.9852	77
thymine	GC	0.0038	15.7	0.9854	71
	B-G	0.0102	12.8	0.9815	115
	N-G	0.0090	12.2	0.9993	106
cytosine	GC	0.0055	9.3	0.9832	114
	B-G	0.0252	13.4	0.9944	133
	N-G	0.0048	3.4	0.5993	114

## References

[b1] Wang X. W. . Heteroatom-doped graphene materials: syntheses, properties and applications. Chem Soc Rev 43, 7067–7098 (2014). 24954470 10.1039/c4cs00141a

[b2] Liu H. T., Liu Y. Q. & Zhu D. B. Chemical doping of graphene. J Mater Chem 21, 3335–3345 (2011).

[b3] Wang X. R. . N-Doping of Graphene Through Electrothermal Reactions with Ammonia. Science 324, 768–771 (2009). 19423822 10.1126/science.1170335

[b4] Lazar P., Zboril R., Pumera M. & Otyepka M. Chemical nature of boron and nitrogen dopant atoms in graphene strongly influences its electronic properties. Phys Chem Chem Phys 16, 14231–14235 (2014). 24912566 10.1039/c4cp01638f

[b5] Ambrosi A., Poh H. L., Wang L., Sofer Z. & Pumera M. Capacitance of p-and n-Doped Graphenes is Dominated by Structural Defects Regardless of the Dopant Type. Chemsuschem 7, 1102–1106 (2014). 24591401 10.1002/cssc.201400013

[b6] Choi C. H., Chung M. W., Kwon H. C., Park S. H. & Woo S. I. B. N- and P, N-doped graphene as highly active catalysts for oxygen reduction reactions in acidic media. J Mater Chem A 1, 3694–3699 (2013).

[b7] Lv R. T. & Terrones M. Towards new graphene materials: Doped graphene sheets and nanoribbons. Mater Lett 78, 209–218 (2012).

[b8] Panchokarla L. S. . Synthesis, Structure, and Properties of Boron- and Nitrogen-Doped Graphene. Adv Mater 21, 4726–4730 (2009).

[b9] Wang Y., Shao Y. Y., Matson D. W., Li J. H. & Lin Y. H. Nitrogen-Doped Graphene and Its Application in Electrochemical Biosensing. ACS Nano 4, 1790–1798 (2010). 20373745 10.1021/nn100315s

[b10] Pumera M. Heteroatom modified graphenes: electronic and electrochemical applications. J Mater Chem C 2, 6454–6461 (2014).

[b11] Xue Y., Wu B., Bao Q. & Liu Y. Controllable Synthesis of Doped Graphene and Its Applications. Small 10, 2975–2991 (2014). 24715648 10.1002/smll.201400706

[b12] Poh H. L., Simek P., Sofer Z., Tomandl I. & Pumera M. Boron and nitrogen doping of graphene via thermal exfoliation of graphite oxide in a BF3 or NH3 atmosphere: contrasting properties. J Mater Chem A 1, 13146–13153 (2013).

[b13] Sheng Z.-H. . Electrochemical sensor based on nitrogen doped graphene: Simultaneous determination of ascorbic acid, dopamine and uric acid. Biosens Bioelectron 34, 125–131 (2012). 22342696 10.1016/j.bios.2012.01.030

[b14] Yang G.-H. . Microwave-assisted synthesis of nitrogen and boron co-doped graphene and its application for enhanced electrochemical detection of hydrogen peroxide. RSC Adv 3, 22597–22604 (2013).

[b15] Hui K. H., Ambrosi A., Sofer Z., Pumera M. & Bonanni A. The dopant type and amount governs the electrochemical performance of graphene platforms for the antioxidant activity quantification. Nanoscale 7, 9040–9045 (2015). 25920751 10.1039/c5nr01045d

[b16] Zhang Y. X., Zhang J. & Su D. S. Substitutional Doping of Carbon Nanotubes with Heteroatoms and Their Chemical Applications. Chemsuschem 7, 1240–1250 (2014). 24678055 10.1002/cssc.201301166

[b17] Deng C. . Electrochemical oxidation of purine and pyrimidine bases based on the boron-doped nanotubes modified electrode. Biosens Bioelectron 31, 469–474 (2012). 22154402 10.1016/j.bios.2011.11.018

[b18] Goh M. S., Bonanni A., Ambrosi A., Sofer Z. & Pumera M. Chemically-modified graphenes for oxidation of DNA bases: analytical parameters. Analyst 136, 4738–4744 (2011). 21956120 10.1039/c1an15631d

[b19] Toh R. J., Bonanni A. & Pumera M. Oxidation of DNA bases is influenced by their position in the DNA strand. Electrochem Commun 22, 207–210 (2012).

[b20] Tan S. M., Poh H. L., Sofer Z. & Pumera M. Boron-doped graphene and boron-doped diamond electrodes: detection of biomarkers and resistance to fouling. Analyst 138, 4885–4891 (2013). 23817573 10.1039/c3an00535f

[b21] Akhavan O., Ghaderi E., Hashemi E. & Rahighi R. Ultra-sensitive detection of leukemia by graphene. Nanoscale 6, 14810–14819 (2014). 25358266 10.1039/c4nr04589k

[b22] Akhavan O., Ghaderi E. & Rahighi R. Toward Single-DNA Electrochemical Biosensing by Graphene Nanowalls. ACS Nano 6, 2904–2916 (2012). 22385391 10.1021/nn300261t

[b23] Akhavan O., Ghaderi E., Rahighi R. & Abdolahad M. Spongy graphene electrode in electrochemical detection of leukemia at single-cell levels. Carbon 79, 654–663 (2014).

[b24] Hummers W. S. & Offeman R. E. Preparation of Graphitic Oxide. J Am Chem Soc 80, 1339–1339 (1958).

[b25] Wang L., Sofer Z., Luxa J. & Pumera M. Nitrogen doped graphene: influence of precursors and conditions of the synthesis. J Mater Chem C 2, 2887–2893 (2014).

[b26] Staudenmaier L. Verfahren zur Darstellung der Graphitsäure. Ber Dtsch Chem Ges 31, 1481–1487 (1898).

[b27] Wang L., Sofer Z., Simek P., Tomandl I. & Pumera M. Boron-Doped Graphene: Scalable and Tunable p-Type Carrier Concentration Doping. J Phys Chem C 117, 23251–23257 (2013).

[b28] Konopka S. J. & McDuffie B. Diffusion Coefficients of Ferricyanide and Ferrocyanide Ions in Aqueous Media, Using Twin-Electrode Thin-Layer Electrochemistry. Anal Chem 42, 1741–1746 (1970).

[b29] Detection Limits-XPS International LLC, http://www.xpsdata.com/Technique_limits_p1.pdf (2016).

[b30] Ferrari A. C. Raman spectroscopy of graphene and graphite: Disorder, electron-phonon coupling, doping and nonadiabatic effects. Sol State Commun 143, 47–57 (2007).

[b31] Banks C. E., Moore R. R., Davies T. J. & Compton R. G. Investigation of modified basal plane pyrolytic graphite electrodes: definitive evidence for the electrocatalytic properties of the ends of carbon nanotubes. Chem Commun, 1804–1805 (2004). 10.1039/b406174h 15306892

[b32] McCreery R. L., Cline K. K., McDermott C. A. & McDermott M. T. Control of Reactivity at Carbon Electrode Surfaces. Colloids Surf, A 93, 211–219 (1994).

[b33] Robinson R. S., Sternitzke K., McDermott M. T. & McCreery R. L. Morphology and Electrochemical Effects of Defects on Highly Oriented Pyrolytic-Graphite. J Electrochem Soc 138, 2412–2418 (1991).

[b34] Compton R. G. & Banks C. E. Understanding Voltammetry. 2nd edn (Imperial College Press, 2011).

[b35] Salimi A., Banks C. E. & Compton R. G. Abrasive immobilization of carbon nanotubes on a basal plane pyrolytic graphite electrode: application to the detection of epinephrine. Analyst 129, 225–228 (2004). 14978524 10.1039/b315877b

[b36] Desimoni E. & Brunetti B. Presenting Analytical Performances of Electrochemical Sensors. Some Suggestions. Electroanalysis 25, 1645–1651 (2013).

[b37] Miller J. C. & Miller J. N. Statistics and Chemometrics for Analytical Chemistry. 5th edn (Pearson Prentice Hall, 2005).

[b38] Currie L. A. Detection: International update, and some emerging di-lemmas involving calibration, the blank, and multiple detection decisions. Chemometrics and Intelligent Laboratory Systems 37, 151–181 (1997).

