# Supplementary material for: Doped Graphene for DNA Analysis: the Electrochemical Signal is Strongly Influenced by the Kind of Dopant and the Nucleobase Structure
Source: Sci Rep. 2016 Sep 14;6:33046. doi: 10.1038/srep33046 (PMC5022011; doi:10.1038/srep33046)
Supplement: Supplementary Information [file srep33046-s1.pdf]

Supporting Information

Doped Graphene for DNA Analysis: the Electrochemical Signal is Strongly Influenced by the Kind of Dopant and the Nucleobase Structure

Huidi Tian,[a] Lu Wang,[a] Zdenek Sofer,[b] Martin Pumera,[a] Alessandra Bonanni[a]*

[a]Division of Chemistry & Biological Chemistry, School of Physical and Mathematical Sciences, Nanyang Technological University, Singapore 637371

[b]Department of Inorganic Chemistry, Institute of Chemical Technology, 166 28 Prague 6, Czech Republic

Fax: (65) 6791-1961

Email: a.bonanni@ntu.edu.sg

*Author for correspondence

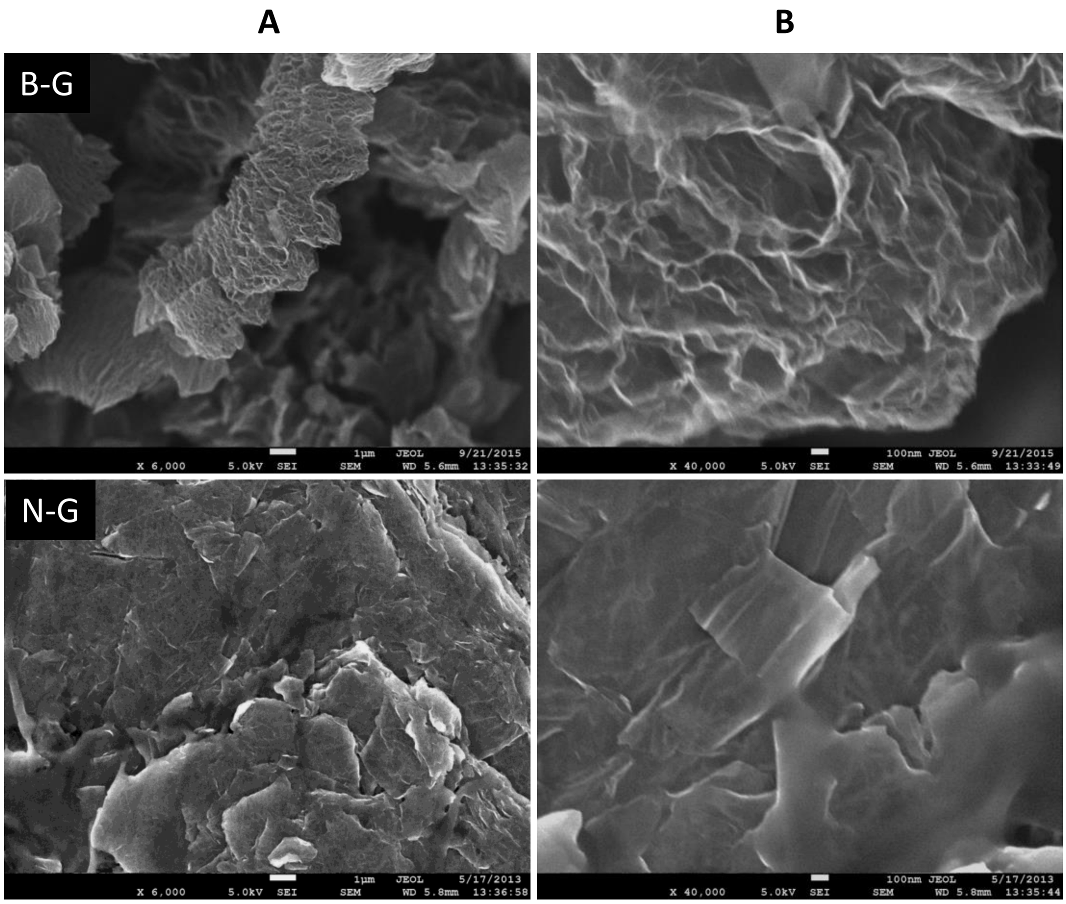

Figure S1. Scanning electron micrographs of boron-doped graphene (B-G) and nitrogen-doped graphene (N-G). A. Magnification at 6000x; scale bar of 1μm; B. Magnification at 40000x; scale bar of 100 nm.

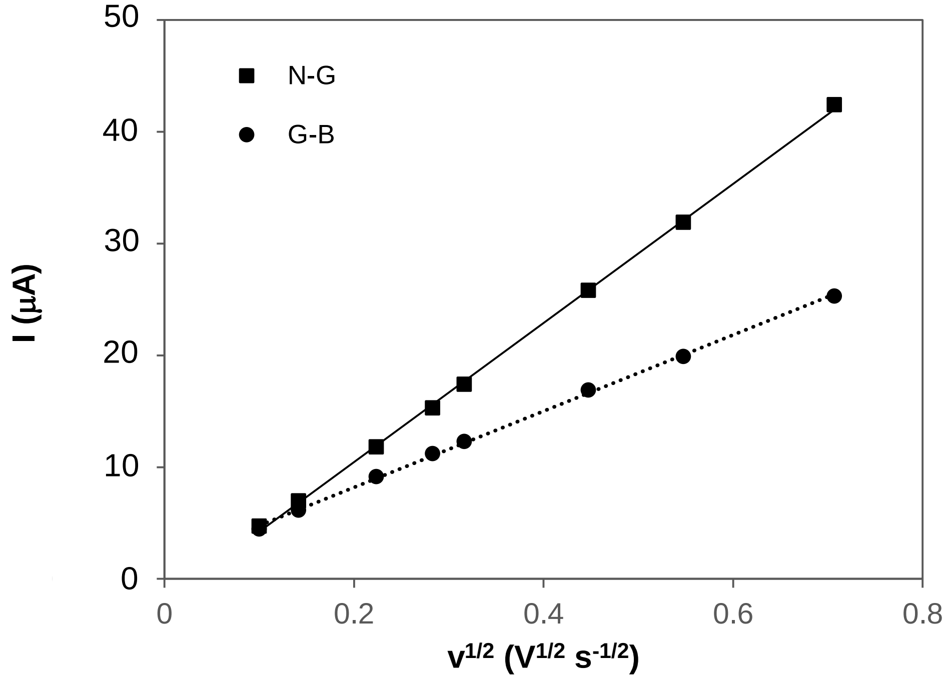

Figure S2. Scan rate study for the determination of electroactive surface area. Peak currents are plotted versus square root of scan rate. Measurements were performed in 1 mM $K_3[Fe(CN)_6]$ in 0.1 M KCl.

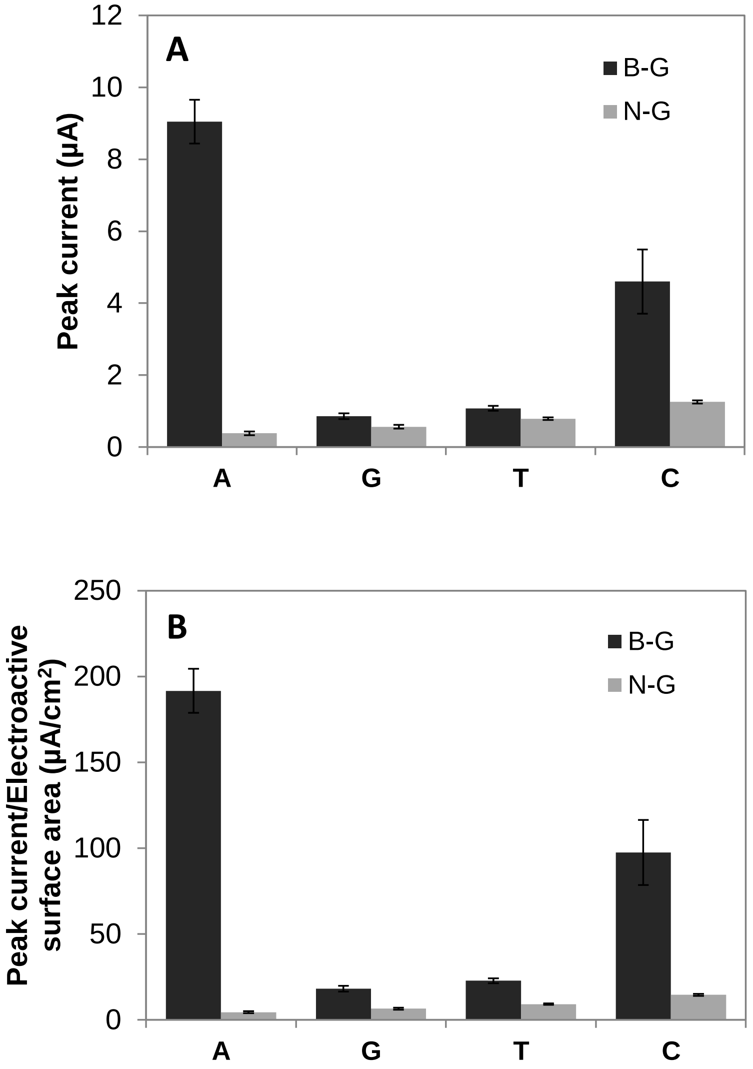

Figure S3. Electrochemical oxidation signal of DNA nucleobases adenine (A), guanine (G), thymine (T) and cytosine (C) on boron-doped graphene (B-G) platform and nitrogen-doped graphene (N-G) platform. Part A: signal represented as peak current (µA). Part B: signal represented as current density (µA/cm^2). Concentrations of DNA bases: adenine 50 µM; guanine 9 µM; thymine 100 nM; cytosine 270 nM.

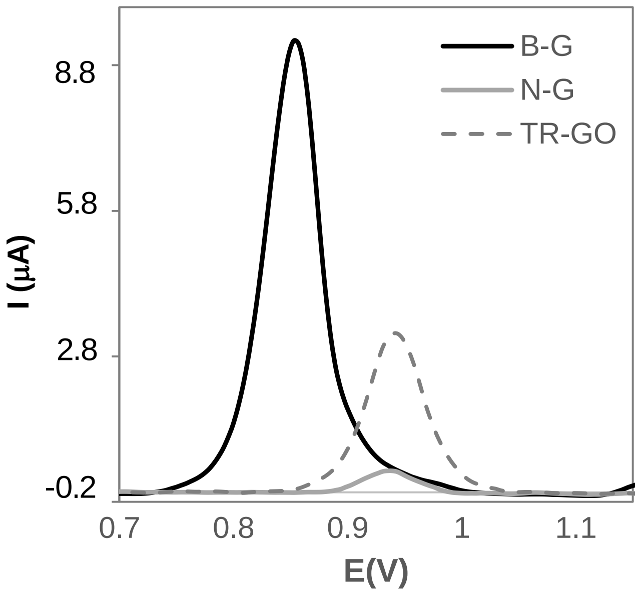

Figure S4. DPV study of adenine on B-G, N-G and undoped graphene (TR-GO) modified electrodes in 50 mM phosphate buffer solution (pH 7.2). Conditions: adenine concentration at 50 μM.

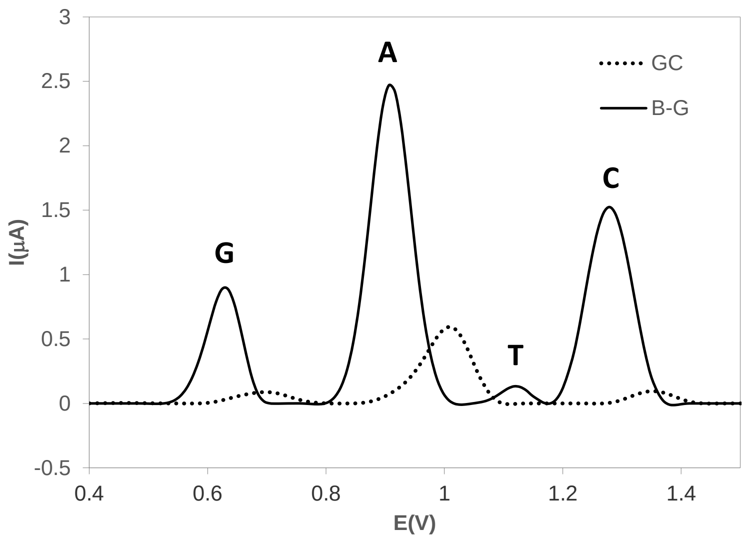

Figure S5. DPV study of adenine, guanine, thymine and cytosine on GC (dotted line) and B-G modified electrode (continuous line) in 50 mM phosphate buffer solution (pH 7.2).

Table S1. Stability study for adenine oxidation on boron-doped graphene platform (B-G). Conditions: adenine concentration 50 µM.

N. of scans	Peak current (µA)	% Initial signal
5	7.70	89
10	6.96	80
15	5.82	67
20	5.56	64

Table S2. Linearity of response (R^2) for scan rate study of DNA nucleobases on B-G and N-G materials. Results are reported for liner curves obtained by plotting either peak intensity (*I*) vs square root of scan rate ($v^{1/2}$) or peak intensity (*I*) vs scan rate (*v*). Scan rates were the following: 25, 50, 75, 100, 125, 150, 200 mV.

	R^2	
	I vs $v^{1/2}$	I vs v
**B-G**		
Adenine	0.9465	0.9900
Guanine	0.8606	0.8676
Thymine	0.9066	0.9709
Cytosine	0.9094	0.9577
**N-G**		
Adenine	0.8090	0.8994
Guanine	0.8517	0.8983
Thymine	0.9515	0.9560
Cytosine	0.9535	0.9862
